# Preliminary Results on the Long-Term Effects of Dextromethorphan on MDMA-Mediated Serotonergic Deficiency and Volumetric Changes in Primates Based on 4-[^18^F]-ADAM PET/MRI

**DOI:** 10.3389/fnins.2022.837194

**Published:** 2022-05-19

**Authors:** Skye Hsin-Hsien Yeh, Yu-Yeh Kuo, Wen-Sheng Huang, Chuang-Hsin Chiu, Tsung-Hsun Yu, Leo Garcia Flores II, Chi-Jung Tsai, Cheng-Yi Cheng, Kuo-Hsing Ma

**Affiliations:** ^1^Brain Research Center, National Yang Ming Chiao Tung University, Taipei, Taiwan; ^2^School of Medicine, National Defense Medical Center, Taipei, Taiwan; ^3^Department of Nursing, Hsin-Sheng College of Medical Care and Management, Taoyuan, Taiwan; ^4^Department of Nuclear Medicine, Cheng-Hsin General Hospital, Taipei, Taiwan; ^5^Department of Nuclear Medicine, Taipei Medical University Hospital, Taipei, Taiwan; ^6^Department of Nuclear Medicine, Tri-Service General Hospital, Taipei, Taiwan; ^7^Radiomedix, Inc., Houston, TX, United States; ^8^Department of Biology and Anatomy, National Defense Medical Center, Taipei, Taiwan

**Keywords:** 4-[^18^F]-ADAM PET, MDMA-induced serotonergic deficiency, SERT reversibility, volumetric changes, neuroprotective effects of dextromethorphan

## Abstract

Alterations to the serotonergic system due to 3,4-methylenedioxymethamphetamine (MDMA) (ecstasy) consumption have been extensively documented. However, knowledge of the reversibility of these neurotoxic effects based on *in vivo* evaluations of serotonin transport (SERT) availability remains limited. This study aimed to evaluate the long-term neurotoxicity of MDMA after 66 months abstinence and explored whether Dextromethorphan, a non-competitive *N*-methyl-*D*-aspartate (NMDA) receptor, could attenuate MDMA-induced neurotoxicity using 4-[^18^F]-ADAM, an imaging ligand that selectively targets SERT, with positron emission tomography technology (PET). Nine monkeys (*Macaca cyclopis*) were used in this study: control, MDMA, and DM + MDMA. Static 4-[^18^F]-ADAM PET was performed at 60 and 66 months after drug treatment. Serotonin transport (SERT) availability was presented as the specific uptake ratios (SURs) of 4-[^18^F]-ADAM in brain regions. Voxel-based region-specific SERT availability was calculated to generate 3D PET/MR images. Structural Magnetic Resonance Imaging (MRI) volumetric analysis was also conducted at 60 months. Significantly decreased 4-[^18^F]-ADAM SURs were observed in the striatum and thalamus of the MDMA group at 60 and 66 months compared to controls; the midbrain and frontal cortex SURs were similar at 60 and 66 months in the MDMA and control groups. All eleven brain regions showed significantly lower (∼13%) self-recovery rates over time; the occipital cortex and cingulate recovered to baseline by 66 months. DM attenuated MDMA-induced SERT deficiency on average, by ∼8 and ∼1% at 60 and 66 months, respectively; whereas significant differences were observed between the thalamus and amygdala of the MDMA and DM + MDMA groups at 66 months. Compared to controls, the MDMA group exhibited significantly increased (∼6.6%) gray matter volumes in the frontal cortex, occipital cortex, caudate nucleus, hippocampus, midbrain, and amygdala. Moreover, the gray matter volumes of the occipital cortex, hippocampus and amygdala correlated negatively with the 4-[^18^F]-ADAM SURs of the same regions. DM (*n* = 2) did not appear to affect MDMA-induced volumetric changes. The 4-[^18^F]-ADAM SURs, lower self-recovery rate and increased volumetric values indicate the occipital cortex, hippocampus and amygdala still exhibit MDMA-induced neurotoxicity after 66 months’ abstinence. Moreover, DM may prevent MDMA-induced serotonergic deficiency, as indicated by increased 4-[^18^F]-ADAM SURs and SERT availability, but not volumetric changes.

## Introduction

3,4-Methylenedioxymethamphetamine (MDMA), commonly known as ecstasy or molly, is a synthetic drug that acts as a stimulant, hallucinogen, and entactogen. MDMA is used recreationally for its mild hallucinogenic and stimulant properties, as well as its ability to increase emotional closeness (Lyles and Cadet, 2003). However, studies have also demonstrated the effectiveness of MDMA might serve a potential treatment for anxiety in people with post-traumatic stress disorder (PTSD) and terminal illnesses (Morgan, 2020).

3,4-Methylenedioxymethamphetamine boosts the activity of three neurotransmitters: dopamine, serotonin (Schmidt et al., 1987; Gough et al., 1991), and norepinephrine (Rothman et al., 2001). The drug enhances synaptic release of these neurotransmitters (Sabol and Seiden, 1998) and/or blocks their reuptake (Berger et al., 1992; Verrico et al., 2008), resulting in increased levels of the neurotransmitters within the synaptic cleft, which in turn affects mood, energy levels, appetite, trust, sexual activity, emotions, and sleep. MDMA also carries serious risks, including hyperthermia, cardiovascular effects, mental impairment, risky behavior, and fatal overdose (Bolla et al., 1998).

While the recreational effects of MDMA usually last for about 3–6 h, the half-life of MDMA is 8–9 h (Freye, 2009). De La Torre et al. (2000) found the peak effects of MDMA are observed within the first 1 and 2 h, and decrease around 4–6 h after taking the drug (De La Torre et al., 2000). Research in rodents has shown that repeated administration of moderate to high doses of MDMA induces long-lasting sensitization of noradrenergic and serotonergic neurons, which correlates with behavioral sensitization (Kirilly, 2010) and selective ablation of serotonergic axon terminals in the forebrain (O’hearn et al., 1988). MDMA-induced 5-HT neural injury in non-human primates lasted for at least 7 years, and may well be permanent (Hatzidimitriou et al., 1999). Several factors are known to influence the recovery of 5-HT axons after administration of MDMA, including the distance between the affected axon terminal field and the rostral raphe nuclei, the degree of initial 5-HT axonal injury, and possibly the proximity of the damaged 5-HT axons to myelinated fiber tracts (Hatzidimitriou et al., 1999; Chiu et al., 2015; Vegting et al., 2016).

The SERT functional imaging have been employed for *in vivo* using single photon emission computed tomography (SPECT) or positron emission tomography (PET) to further explore the effects of MDMA on the serotonergic system (Skye and Hwang, 2016). Using [^11^C]-(+)-McN5652 PET, Mccann et al. (2005) observed decreased global and local binding to SERT in 23 previous ecstasy users compared to 19 non-MDMA controls (Mccann et al., 2005). Semple et al. (1999) used [^123^I]-2β-carbomethoxy-3β-(4-fluorophenyl)tropane ([^123^I]-β-CIT) SPECT and detected a cortical reduction in cerebral SERTs in long-term MDMA users compared with MDMA-naive subjects who use other drugs (Semple et al., 1999). Buchert et al. (2003) demonstrated that the distribution volume ratios (DVR) in the mesencephalon and thalamus were significantly lower in current ecstasy users compared to drug-naive control subjects, and concluded that ecstasy-induced protracted alterations to the brain serotonergic system last several weeks (Buchert et al., 2003).

Dextromethorphan (DM, 3-methoxy-17-methylmorphinan), a non-competitive *N*-methyl-*D*-aspartate (NMDA) receptor antagonist, has been widely used as an antitussive agent. DM has been suggested to reduce neuronal damage and modulate pain sensations *via* non-competitive antagonism of excitatory amino acids (EAAs) (Siu and Drachtman, 2007). Excitotoxicity is a phenomenon that describes the toxic actions of excessive excitatory neurotransmitters, primarily the EAA glutamate, that lead to exacerbated or prolonged activation of receptors, which starts a cascade of neurotoxicity that ultimately leads to the loss of neuronal function and cell death (Armada-Moreira et al., 2020; Suzuki et al., 2021). In addition to its therapeutic effects in terms of antitussive activity (Craviso and Musacchio, 1983), cancer pain relief (Weinbroum et al., 2000), and methotrexate toxicity (Kishi et al., 2000), DM has also been used as neuroprotective agent for seizures (Schmitt et al., 1994), cerebral ischemia (Lo and Steinberg, 1991) and Parkinson’s disease (Liu et al., 2019).

As described above, in the last two decades, a number of SERT imaging agents have become available for human SPECT or PET studies, and some of these agents labeled with ^11^C or ^123^I have been used for MDMA-related neuroimaging. These include [^123^I]I-β-CIT (Laruelle et al., 1994) and [^123^I]I-ADAM (Newberg et al., 2004) for SPECT, and [^11^C]-(+) McN5652 (Buck et al., 2000), [^11^C]DASB (Ginovart et al., 2001) and [^11^C]AFM (Naganawa et al., 2013) for PET.

However, ^18^F has some advantages over ^11^C, notably that the ^18^F-labeled radioligands can be transported offsite if a cyclotron is not available. The most important advantages of PET imaging are its much higher sensitivity and better contrast and spatial resolution compared to SPECT (Rahmim and Zaidi, 2008). Therefore, a number of ^18^F-labeled SERT imaging agents such as S-[^18^F]fluoroethyl)-(+)-McN5652 (Suehiro et al., 1996), S-[^18^F]fluoromethyl)-(+)-McN5652 (Brust et al., 2003), and [^18^F]F-ACF (Oya et al., 2002) have been reported. Based on its structure and our experience with [^123^I]I-ADAM, our group and others made efforts to develop ^18^F-labeled SERT imaging agents, including 4-[^18^F]F-ADAM (Shiue et al., 2003; Peng et al., 2008; Huang et al., 2009), 5-[^18^F]F-ADAM (Fang et al., 2004), and [^18^F]F-AFM (Huang et al., 2005). The structures of [^123^I]I-ADAM and 4-[^18^F]F-ADAM are shown in Supplementary Figure 1.

Among these ^18^F-labeled SERT imaging agents, S-[^18^F]fluoromethyl)-(+)-McN5652 has been studied in humans and shown to be suitable for *in vivo* quantification of SERT with PET (Hesse et al., 2012), and 4-[^18^F]F-ADAM developed by our group has been synthesized and evaluated in translational studies of its specificity for SERT in rodents (Ma et al., 2009) and primates (Huang et al., 2010). Moreover, the safety and high specificity of 4-[^18^F]-ADAM for SERT have also been demonstrated in human studies (Huang et al., 2013).

With 4-[^18^F]-ADAM PET, we also reported that MDMA induced neurite damage and neuron death in serotonergic neurons *in vitro* (Li et al., 2016). MDMA-treated rats exhibited reduced SERT availability in the midbrain and thalamus at 2 weeks, and these changes were associated with depressive-like behaviors (Shih et al., 2016).

In parallel to developing 4-[^18^F]-ADAM, in order to investigate the topic of MDMA-induced SERT deficiency and explore whether DM exerts a neuroprotective effect, we performed SERT imaging using [^123^I]-ADAM SPECT in the primate brain (Ma et al., 2009). We observed the MDMA-induced decrease in central SERT levels persisted for over 4 years. The [^123^I]-ADAM signals were significantly lower in the brains of the MDMA group than the control group, indicating the MDMA-treated monkeys had lower brain SERT levels. We also reported that DM exerts a protective effect against MDMA-induced serotonergic aberrations (Ma et al., 2016).

In the present study, by using [^18^F]-4-ADAM PET/CT/MR, we further evaluated SERT availability, self-recovery rate, and volumetric changes in various brain regions of MDMA-exposed primates 6.5 years after administration of the drug using and also assessed the neuroprotective effects of DM. To extend our previous [^123^I]-ADAM SPECT results, the motivation of this study was to (1) to gain a better understanding of the extended long-term SERT deficiency post-MDMA up to 66 months; (2) to emphasize that certain specific regions; i.e., 3D detailed regional-specific SERT PET imaging post-MDMA, is superior to SPECT images; and (3) to highlight that current state-of-the-art PET and MRI scanners can greatly benefit from improvements in innovative image reconstruction algorithms; i.e., MDMA-induced alterations of structural MRI volumetric analysis *vs.* functional PET SERT activity.

## Materials and Methods

### Radiosynthesis of 4-[^18^F]-ADAM

4-[^18^F]-ADAM, a specific radioligand for SERT, was synthesized using an automated synthesis device, as previously reported (Peng et al., 2008). In brief, the precursor 4-[^18^F]-ADAM in 0.5 mL of dimethyl sulfoxide was added to dried potassium [^18^F]-fluoride/Kryptofix 2.2.2 residue and reduced with NaBH_4_/Cu(OAc)_2_. Purification by high-performance liquid chromatography produced the desired compound with a radiochemical yield of approximately 3% at the end of synthesis (EOS) in a synthesis period of 120 min. The EOS of 4-[^18^F]-ADAM can be increased to approximately 15% by using a different precursor and manual synthesis (Huang et al., 2009). The chemical and radiochemical purities were >95%, and the specific activity was >3 Ci μmol^–1^ (111 GBq μmol^–1^).

### Experimental Animals

Nine Formosan rock monkeys (*Macaca cyclopis*) aged 4–6-years-old were assessed in this study (Table 1). The animals were housed separately in a facility accredited by the Association for Assessment and Accreditation of Laboratory Animal Care International (AAALAC International), at National Defense Medical Center. All experiments were performed following National Defense Medical Center’s guidelines for conducting experiments in non-human primates under an Institutional Animal Care and Use Committee approved research protocol (IAUIC No 12-188).

**TABLE 1:** Summary of the characteristics of the animals before PET imaging with [^18^F]-4-ADAM.

Animals	Male	Female	All
Sex, *n*	6	3	9
Weight (Kg)	7.78 ± 1.57	5.10 ± 0.52	6.89 ± 1.83
Inject dose (mCi)	5.17 ± 0.64	5.09 ± 0.57	5.14 ± 0.61
Inject dose (mBq)	191.15 ± 23.92	196.83 ± 24.47	193.04 ± 24.49
Inject dose (mBq/Kg)	25.37 ± 5.63	39.06 ± 7.62	29.94 ± 9.04

### Study Design and Drug Treatment

**FIGURE 1:**
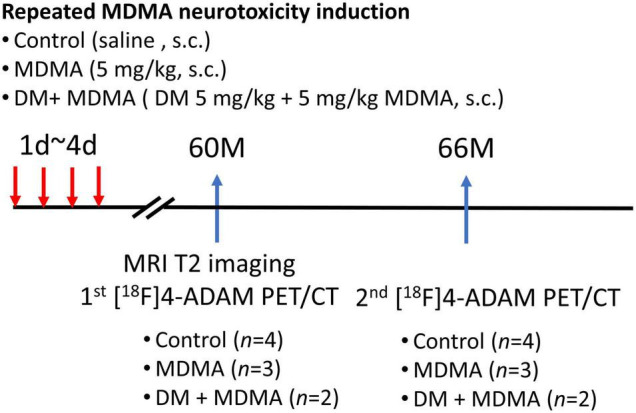
Flowchart of the study design. A total of nine monkeys were randomized into three groups: control, MDMA, and DM + MDMA. The drugs were administered between day 1 and day 4 (twice a day for four consecutive days). MRI imaging was performed at 60 months and PET/CT imaging was performed at 60 and 66 months.

The experimental design is illustrated in Figure 1. Nine monkeys (*Macaca cyclopis*) were used in this study: control, MDMA (5 mg/kg, twice a day for four consecutive days), and DM co-administered with MDMA (MDMA 5 mg/kg with DM 5 mg/kg, twice a day for four consecutive days). The animals used in this study were the same animals used in our previous work, in which we monitored MDMA-induced serotonergic aberrations up to 54 months using [^123^I]ADAM SPECT (Ma et al., 2016); the current study was a follow-up extension up to 66 months based on 4-[^18^F]ADAM PET/CT/MRI imaging.

3,4-Methylenedioxymethamphetamine was provided by the Investigation Bureau of Taiwan and DM (Sigma-Aldrich) challenge studies were used to explore the protective effects of DM against MDMA neurotoxicity. In the MDMA challenge studies, the dose regimen of MDMA was modified from the dose used in a previous study (Szabo et al., 2002). The monkeys were subcutaneously injected with 5 mg/kg MDMA twice a day for four consecutive days to damage the serotonergic system. This particular dosage regimen has been proven to lead to moderate to severe damage in the serotonergic system (Ricaurte et al., 1988; Hatzidimitriou et al., 1999).

Brain 4-[^18^F]-ADAM PET studies were performed 60 and 66 months after the neurotoxin treatment in the MDMA-treated group, MDMA + DM co-administration group, and the control group. All animals in each group were scanned two times to access SERT availability and the progression and recovery of neurotoxicity of the serotonergic systems. The interval between each PET imaging session was 6 months. The average body weight of animal over study period is shown in Table 2.

**TABLE 2:** Bioinformation on animal body weight during the study period.

	Average body weight of monkey over study period				
	
Groups	60 M	*n*	>66 M	*n*	72 M
Control	5.7 ± 1.7	4	6.1 ± 1.5	4	8.2 ± 1.8
MDMA	7.8 ± 2.6	3	8.4 ± 2.1	3	6.7 ± 0.6
DM + MDMA	7.1 ± 0.7	2	7.1 ± 0.8	2	7.6 ± 0.9

*There were only two animals in MDMA + DM group because one subject was withdrawn due to incomplete data.*

### Positron Emission Tomography Imaging and Data Acquisition

Prior to imaging, the animals were fasted overnight with free access to water. The animals were immobilized with Zoletil™ (4 mg/kg, I.M. injection), and anesthesia was maintained with passive inhalation of oxygen containing 1.8% isoflurane at a rate of 2 L/min (to maintain >95% oxygen saturation). If necessary, Atropine (0.05 mg/kg I.M. injection) was administered to reduce salivary and bronchial secretions during Zoletil™ anesthesia to avoid excessive swallowing, reflexes, and accumulation of saliva during the experiment, which may cause vomiting and nausea. Intravenous infusion of 0.9% NaCl at a rate of 5 mL/kg per hour *via* the cephalic vein was used to maintain hydration and for radiotracer administration.

Positron emission tomography/CT measurements were performed in 3D mode on a Biogragh 2 scanner (Biograph Duo, Siemens), which includes a dual slice detector CT scanner (Somatom Emotion; Siemens Medical Systems). This scanner has a transverse FOV of 58.5 cm, an axial FOV of 15.5 cm and a spatial resolution of 4.8 mm. Each subject was carefully placed on the motorized adjustable bed of the scanner and their head was immobilized using a cradle. Before the emission scan, a scout scan was performed to define the imaging field, followed by a low-dose CT scan (130 kVp, 50 mAs, 0.8 s tube rotation, 4-mm slice collimation, pitch 3), followed by a static PET scan in 3D mode at 120–150 min after intravenous bolus injection of 192.48 ± 23.93 MBq (5.13 ± 0.59 mCi) of 4-[^18^F]-ADAM. Emission images were reconstructed in a 512 × 512 × 64 matrix with a pixel size of 0.519 mm × 0.519 mm × 2.4 mm using OSEM (six iterations and 16 subsets) using a Gaussian filter with a FWHM of 3 mm. All reconstructed PET images were corrected for attenuation using the CT images. The CT images also served as anatomic references for registering the PET data when defining ROIs.

### Magnetic Resonance Imaging Protocol

T2-weighted Magnetic Resonance (MR) imaging was performed using a 3.0 Tesla GE SIGNA 450 system. After three-plane tri-pilot imaging, 22 contiguous coronal, sagittal and horizontal T2WIs were acquired using a fast spin echo sequence with a TR/TE of 3,000/100 ms, echo train length of 8, NEX of 4, matrix size of 256 × 256, FOV of 20 × 20 mm^2^, slice thickness (SLTH) of 4 mm, flip angle of 90°, bandwidth of 50.0 kHz, and acquisition time of 6 min 44 s. All animals were imaged using the same MR parameters. After the image acquisition was complete, the images were transferred to a stand-alone personal computer and analyzed using N.I.H. Image 1.52 software. The MR images were resliced, resized, and co-registered to all corresponding PET images in the plane parallel to the canthomeatal line (CML).

### Positron Emission Tomography/Magnetic Resonance Data Analyses

We delineated the region of interest (ROIs) using an automated anatomical labeling template (monkey atlas) in PNEURO (PMOD version 4.0, PMOD Technologies, Zurich, Switzerland) in order to prevent bias from inter- or intra-rater reliability issues arising from manual delineation. Then, the mean standardized uptake value (SUV) in frontal cortex (FCX), midbrain (MB), thalamus (TH), striatum (STR), and cerebellum (CB) were extracted from unsmoothed SUV images in the standard stereotactic space. The CB expresses very low concentrations of SERT and was used as the reference region (Kish et al., 2005). The cerebellar vermis was excluded and only the posterior half of the cerebellar cortex was delineated.

### Specific Uptake Ratio of 4-[^18^F]-ADAM

The specific binding of 4-[^18^F]-ADAM in SERT-rich regions was semi-quantitatively analyzed and expressed as the specific uptake ratio (SUR). The CB was chosen as the reference region for free and non-specific binding in the brain. The SUR of various ROIs, including the MB, TH, STR, and FCX, were calculated and expressed using Equation (1):


(1) SUR=(C-ROIC/CB)CCB

where C_ROI_ and C_CB_ are the mean radioactivity at “pseudo” equilibrium in the ROIs and CB, respectively.

### Magnetic Resonance Imaging-Based Volumetric Quantification

In addition to the PET data, the MRI images obtained at the 60-month imaging timepoint were reconstructed; brain segment images representing a labeled atlas of the brain structures identified by the segmentation algorithm were created in PNEURO (PMOD version 4.0). Volumetric quantification was performed for the stratum, midbrain, thalamus, frontal cortex, and cerebellum of each subject.

### Statistical Analysis

Data are expressed as mean ± SEM. One-way ANOVA with the *post-hoc* Bonferroni or unpaired Student’s *t*-test were used for statistical evaluation; *p* < 0.05 was defined as statistically significant. Statistical analyses were performed using GraphPad Prism 8 (GraphPad software, La Jolla, CA, United States).

## Results

### Repeated 3,4-Methylenedioxymethamphetamine Administration Induces Long-Lasting Serotonergic Neurotoxicity in the Striatum

**FIGURE 2:**
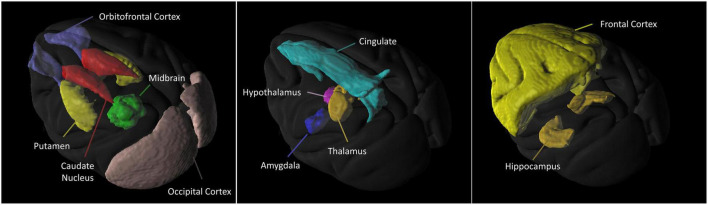
3D 4-[^18^F]-ADAM PET images. Illustration of the location of the brain regions was used to estimate the SERT binding of 4-[^18^F]-ADAM.

**FIGURE 3:**
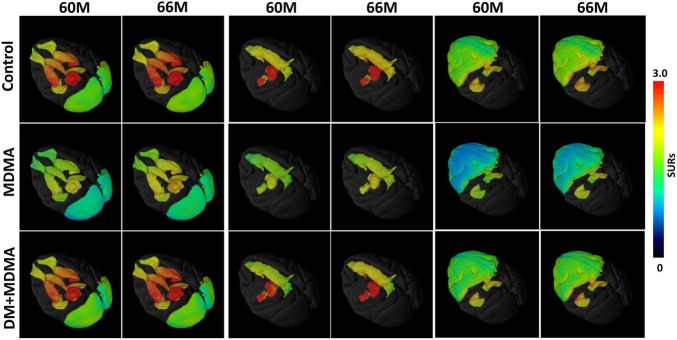
3D PET parametric SUR imaging obtained 120–150 min after intravenous injection of [^18^F]-4-ADAM in different regions. Multiple-assembly brain region (top row); control group (second row); MDMA group (third row); and DM + MDMA group (bottom row). PET images were co-registered to MRI-based atlas to generate 3D PET/MR images (PMOD version 4.0, PMOD Technologies, Zurich, Switzerland).

Figure 2 is the illustration of the location of the brain regions was used to estimate the SERT binding of 4-[^18^F]-ADAM. The 3D PET/MRI brain images shown in Figure 3 present evaluation of the 4-[^18^F]-ADAM uptakes in the control group and at 60 and 66 months after administration of MDMA with or without DM. On average, the midbrain showed the highest uptake of [^18^F]-ADAM during the study period, followed by the thalamus, putamen, caudate nucleus, amygdala, and the other regions. The reference region, the cerebellum, had relatively lower SURs compared to other regions.

**FIGURE 4:**
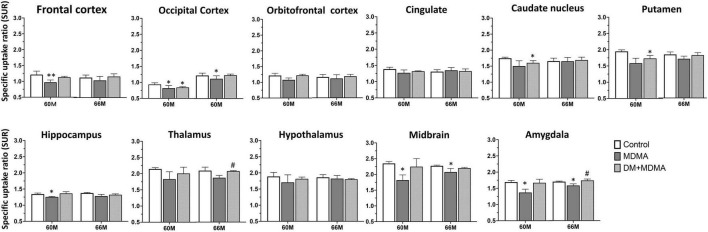
Specific uptake ratios of [^18^F]-4-ADAM PET obtained 120–150 min post-injection for various brain regions in the control, MDMA, and DM + MDMA groups at 60 and 66 months. Data is presented as mean ± SEM; *p < 0.05 compared to control group. #*p* < 0.05 compared to the MDMA group. **p < 0.01.

As shown in Figure 4, lower 4-[^18^F]-ADAM SURs were observed in all regions in the MDMA groups at 60 and 66 months compared to the controls. Significant reductions were detected in the frontal cortex, occipital cortex, hippocampus, midbrain and amygdala (*p* < 0.05). Detailed results of the specific uptake ratio (SURs) *in vivo* are shown in Supplementary Table 1.

### Region-Specific Recovery of Serotonin Transport Availability After 3,4-Methylenedioxymethamphetamine Induction

**FIGURE 5:**
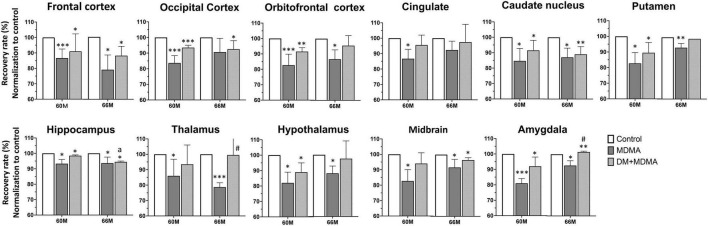
Recovery rate of SERT availability in various brain regions in the control, MDMA, and DM + MDMA groups at 60 and 66 months. Data is presented as mean ± SEM; *p < 0.05, **p < 0.01, ***p < 0.005 compared to the control group; #*p* < 0.05 compared to the MDMA group. *ap* < 0.01 when compared to DM+MDMA at 60 months.

We next evaluated whether SERT availability could recover over time. All regions showed significantly lower self-recovery rates over time after administration of MDMA (*p* < 0.05∼*p* < 0.005), whereas the occipital cortex and cingulate recovered to baseline levels (control group) within 66 months (Figure 5).

### Dextromethorphan Attenuates 3,4-Methylenedioxymethamphetamine-Induced Serotonin Transport Deficiency

Next, we tested the hypothesis that treatment with DM attenuates MDMA-induced SERT deficiency by accelerating the recovery of SERT availability. Compared to the MDMA group, DM attenuated MDMA-induced SERT deficiency from 85.55 ± 5.72% to 93.23 ± 3.48% and 88.91 ± 6.17% to 89.91 ± 7.41% at 60 and 66 months, respectively. There were significant differences between the thalamus and amygdala of the MDMA and DM + MDMA groups at 66 months (#*p* < 0.05, Figure 4). Detailed results of the recovery rate of SERT availability *in vivo* are shown in Supplementary Table 2.

### 3,4-Methylenedioxymethamphetamine Affects Brain Volume, but Dextromethorphan Does Not Attenuate These Changes

**FIGURE 6:**
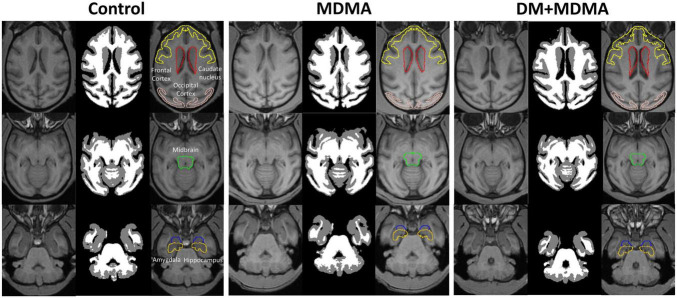
Volumetric differences between the control (Left), MDMA (middle), and DM + MDMA (right) groups. MDMA led to higher gray matter volumes in young primates; DM + MDMA had no effect on the MDMA-induced changes in brain volume.

**FIGURE 7:**
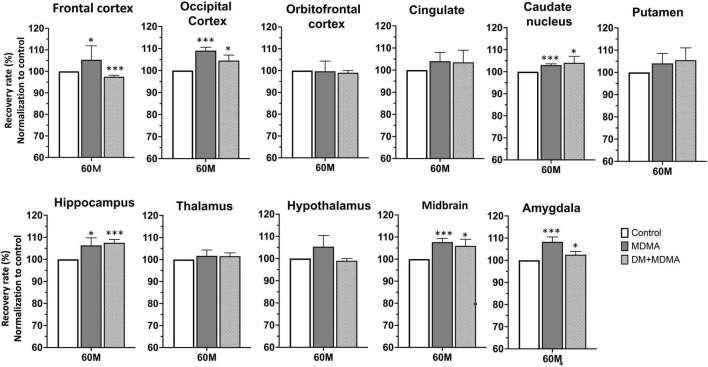
Volumetric analysis of brain MR images. Comparison showing the volumes of the striatum, midbrain, thalamus and frontal cortex in MDMA group were significantly larger (*p* < 0.01∼0.005) compared to controls. Co-administration of DM with MDMA had no significant neuroprotective effect on volumetric values. Data are presented as mean ± SEM; *p < 0.05, ** *p* < 0.01, ***p < 0.005 compared to the control group.

The volumes of the frontal cortex, occipital cortex, caudate nucleus, hippocampus, midbrain and amygdala were significantly increased in the MDMA group compared to controls (*P* < 0.01∼0.005, Figure 6). On average, MDMA increased the brain volume in the regions mentioned above by 6.06 ± 2.18% compared to controls. However, DM had no effect on the changes in volume induced by MDMA (DM + MDMA *vs.* MDMA; Figure 7). Detailed results of the MRI volumetric differences *in vivo* are shown in Table 3.

**TABLE 3:** MRI volumetric ratio.

	MRI volumetric differences					
	
	Volumetric ratio	*p _*vs.*_*_*Con*_	*p _*vs.*_*_*MDMA*_	*r2* value	*p _*vs.*_*_*non–zero*_
**Striatum**					
Control	100.00%			1.0000	
MDMA	121.87 ± 3.95%	***		0.0039	*NS*
DM + MDMA	119.00 ± 6.06%	***	*NS*	1.0000	
All groups				0.7200	***
**Thalamus**					
Control	100.00%			1.0000	
MDMA	121.84 ± 7.86%	**		0.9826	0.0843
DM + MDMA	111.17 ± 10.93%	*NS*	*NS*	1.0000	
All groups				0.4966	*
**Midbrain**					
Control	100.00%			1.0000	
MDMA	135.20 ± 7.73%	***		0.1071	*NS*
DM + MDMA	134.57 ± 12.52%	***	*NS*	1.0000	
All groups				0.5473	*
**Frontal cortex**					
Control	100.00%			1.0000	
MDMA	113.12 ± 7.84%	*		0.8799	*NS*
DM + MDMA	99.94 ± 1.54%	*NS*	*NS*	1.0000	
All groups				0.6938	**

Data are presented as mean ± SEM; *p < 0.05, **p < 0.01, ***p < 0.005 compared to the control group. NS, no significant difference.

**FIGURE 8:**
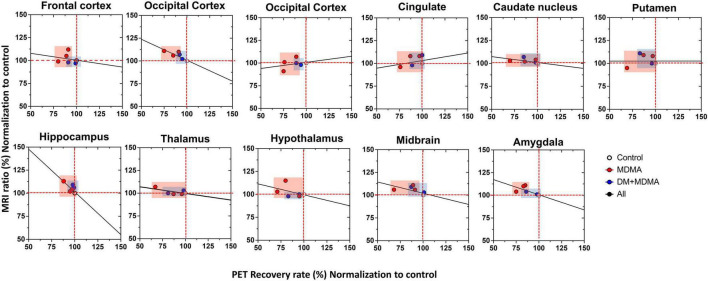
Relationship between the volumetric ratio determined by MRI (*y*-axis) and SERT recovery rate determined by [^18^F]-4-ADAM PET (*x*-axis). Increased brain volume negatively correlated with reduced uptake of [^18^F]-4-ADAM after MDMA administration. Linear correlation coefficients between *X* and *Y* are shown when the two variables ranges; MDMA group-pink block, DM-MDMA-blue block, black line-all grange/group (R^2^-values for each region are shown in Table 4).

Compared to the controls (white circle), MRI brain volume increased post-administration of MDMA, but PET SERT recovery values decreased in all regions (red dot in pink block in Figure 8). In animals co-administered DM, the PET SERT recovery values tended to increase (*x-axis*), but DM had no effect on the brain volume (*y-axis;* blue dot in blue block in Figure 8). Furthermore, after fitting all data sets (black line in Figure 7), the increased brain volumes correlated negatively and significantly with the PET SERT recovery values in the occipital cortex (*R*^2^ = 0.757), hippocampus (*R*^2^ = 0.601; all *P* < 0.05). Detailed results of the *in vivo* PET *vs*. MRI correlations are shown in Table 4.

**TABLE 4:** Simple linear regression model of MRI ratio and PET 4-[^18^F]ADAM recovery rate (R-squared) for the data set of each group or all groups.

	PET *vs.* MRI correlations	
	
Striatum	Slope	*r2* value
Control		1.0000
MDMA	–0.1154	0.0039
DM + MDMA	–6	1.0000
All groups	–1.3096	0.7200
**Thalamus**		
Control		1.0000
MDMA		0.9826
DM + MDMA		1.0000
All groups	–1.17	0.4966
**Midbrain**		
Control		1.0000
MDMA		0.1071
DM + MDMA		1.0000
All groups	–1.5306	0.5473
**Frontal cortex**		
Control		1.0000
MDMA		0.8799
DM + MDMA		1.0000
All groups	–0.72896	0.6938

## Discussion

This *in vivo* functional 4-[^18^F]-ADAM PET/CT/MR imaging study demonstrated that MDMA could induce long-lasting SERT over 66 months in the central nervous system (CNS) of primates. Moreover, the recovery rate of SERT availability and neuroprotective effects of DM were region-specific. These results, particularly after administration of high dosages, are in good agreement with our previous study (Ma et al., 2016) and recent findings (Vegting et al., 2016).

Compared to the aforementioned studies of SERT reversibility in humans after MDMA exposure, we observed some interesting findings in primates. In 2016, we reported that the MDMA-induced decrease in brain SERT levels could persist for over 4 years (48 months) based on the SURs of [^123^I]ADAM SPECT (Ma et al., 2016); we also reported that DM abolished MDMA-induced aberrations in SERT density in several brain regions.

In this study, we extended the follow-up to 66 months. More accurate and detailed 3D PET/MR images revealed the 4-[^18^F]-ADAM PET uptake ratio varied in different brain regions, and we further analyzed the recovery rate of SERT availability to determine self-recovery over time. We also evaluated whether DM attenuates the MDMA-induced reduction in SERT availability or accelerates the recovery rate.

At 60 months post-MDMA exposure (without DM), the mean self-recovery rate of the 11 tested brain regions had reached >88%. At 66 months, the self-recovery rate remained unchanged (89%), suggesting self-recovery had stopped occurring. Significantly lower self-recovery rates for the occipital cortex, hippocampus and amygdala relative to other brain regions tested indicate these regions still exhibit MDMA-induced neurotoxicity after 66 months after last MDMA exposure. These results indicate region-specific recovery of SERT availability after MDMA induction occurs.

Consistent with these conclusions in animals, human studies also revealed significantly lower SERT binding in specific brain areas, predominantly the occipital cortex (*p* < 0.05∼0.0001) (Mccann et al., 1998; Semple et al., 1999; Mccann et al., 2005; De Win et al., 2008; Mccann et al., 2008; Frokjaer et al., 2014), hippocampus (*p* < 0.05∼0.0001) (Mccann et al., 2005, 2008; De Win et al., 2008; Kish et al., 2010; Frokjaer et al., 2014), and thalamus (*p* < 0.05∼ < 0.003) (Mccann et al., 2005; Buchert et al., 2007; De Win et al., 2008).

Moreover, studies that included cognitive performance measures support the imaging findings described above. Reneman et al. (2000) reported higher binding ratios for the post-synaptic 5-HT2A receptors observed using [^123^I]R91150 SPECT were associated with poor memory function, as indicated by a verbal memory test, in MDMA users compared to controls (Reneman et al., 2000). In addition to cortical regions, Bosch et al. (2013) demonstrated significantly decreased regional cerebral brain glucose metabolism (rMRGlu) correlated with poorer verbal learning and delayed recall performance (Bosch et al., 2013). Taken together, this evidence indicates MDMA-induced serotonergic deficiency may be brain region-specific.

In this study, DM attenuated MDMA-induced serotonergic deficiency, and was associated with a reduced density of cerebral SERT; this protective effect was observed up to 66 months later. The protective mechanism of DM may include serotonin reuptake inhibition (Henderson and Fuller, 1992; Gillman, 2005) through its proposed high-affinity binding to the serotonin transporter (Meoni and Bowery, 1997) or inhibition of serotonin (Narita et al., 1995). DM only had a significant neuroprotective effect in the thalamus and amygdala at 66 months after MDMA exposure, but not in the other brain regions tested. This could be due to individual variations and the small sample size (*n* = 2), which resulted in high standard deviations for the MDMA or DM + MDMA group and may have reduced the power to detect significant differences. The variations in the regional distribution of SERT may also help to explain why the protective effects of DM were less pronounced in the tested regions, as larger doses of DM may be required to block the increased number of SERT.

However, studies have also reported that if DM replaces codeine in cough medicines and the approved doses are exceeded, DM produces dissociative effects similar to ketamine and phencyclidine (PCP) (Roy et al., 2015). Therefore, further experiments employing higher doses of DM would help to clarify the relationship between increased SERT availability and the regional alterations in MDMA-induced serotonergic deficiency.

Additionally, it is interesting to note that the brain volumes of the frontal cortex, occipital cortex, caudate nucleus, hippocampus, midbrain, and amygdala increased in the MDMA group compared to controls. Similarly, global reductions in the volumes of gray matter and left orbitofrontal and right occipital regions have been reported in long-term users (Daumann et al., 2011; Koester et al., 2012). Unlike the findings on chronic use (Cowan, 2007; Mueller et al., 2016), Mackey et al. (2014) reported that the gray matter volumes in the vmPFC and insula increased in a population of younger college-aged occasional users of amphetamine-type stimulants (ATS) and cocaine compared to controls (Mackey et al., 2014). They concluded that the increased gray matter volumes in the vmPFC and insula contrasted with the age-related decline in gray matter volume in the healthy developing brain, suggesting that gray matter development may be delayed in young occasional ATS and cocaine users (Mackey et al., 2014).

The orbito- and middle frontal cortex and striatum are important neural structures involved in decision-making. These regions are associated with the reward system and also play a role in addictive disorders (Betzler et al., 2017). One possible explanation for the higher volumes of the frontal cortex, occipital cortex, caudate nucleus, hippocampus, midbrain, and amygdala in the MDMA group could be that acute, repeated low-dose MDMA (5 mg/kg MDMA twice a day for four consecutive days) may lead to selective synaptic reorganization in the ventral striatum and medial frontal cortex (Robinson and Kolb, 2004). Diaz Heijtz et al. (2003) demonstrated that low doses of amphetamine increased spine density and dendritic branching in young rats (Diaz Heijtz et al., 2003). It is possible that the relationship observed between MDMA exposure and increased frontal cortex, occipital cortex, caudate nucleus, hippocampus, midbrain, and amygdala volumes in young primates may be related to excessive proliferation of neuronal processes, associated with structural plasticity, and reflect reorganization of patterns of synaptic connectivity in these neural systems that alters their operation, which may thus contribute to some of the persistent sequela associated with drug use (Robinson and Kolb, 2004).

Taken together, the present study demonstrates that the brain volume values of the occipital cortex, hippocampus and amygdala correlate negatively with the rate of recovery of PET 4-[^18^F]ADAM SERT activity in the same regions. Our findings suggest that MDMA exposure leads to functional abnormalities in a network of brain regions that are important in decision-making processes. In addition, the increased brain volume may compensate for the decreased brain SERT activity in the same regions, and reflect compensatory or neural plasticity in the MDMA-exposed group.

### Limitations

The animals used in this study were the same animals used in our previous work reported in 2016 (Ma et al., 2016); thus, the major drawback is the lack of pre-MDMA baseline scans, although non-MDMA animals were used as controls. Moreover, we did not collect enough data to investigate the links between functional PET SERT activity and structural MRI measures of volumetric values. In addition, most statistical analyses of the DM + MDMA group did not reach statistical significance, although strong decreasing trends were observed. This may be due to the small sample size of this study; further studies with larger sample sizes and additional data are needed to validate our findings.

## Conclusion

The present study examined MDMA-induced serotonergic deficiency in primates at 60 and 66 months after drug administration. 4-[^18^F]-ADAM PET/CT/MRI revealed MDMA-induced serotonergic deficiency may be region-specific, as indicated by the significantly lower 4-[^18^F]-ADAM binding ratios and recovery rates in most brain regions. Unlike findings on chronic use, the increased occipital cortex, hippocampus and amygdala volumes in younger primates exposed to MDMA tended to negatively correlate with the 4-[^18^F]-ADAM PET SURs in the same regions. This may be a compensatory effect for the decreased brain SERT activity in the reward regions, and reflect compensatory or neural plasticity in the MDMA-exposed group. DM appears to exert a neuroprotective effect on SERT activity in the hippocampus and amygdala at 66 months after MDMA exposure, but not against MDMA-induced changes in brain volume. Additional research based on molecular imaging paired with functional neuroimaging, genetics, or pharmacological challenge of the serotonin system is necessary to decipher the link between the serotonergic and cognitive changes induced by MDMA.

## Data Availability Statement

The raw data supporting the conclusions of this article will be made available by the authors, without undue reservation.

## Ethics Statement

This study involving animals was reviewed and approved by the Institutional Animal Care and Use Committee approved research protocol (IAUIC No 12-188). All experiments were performed following National Defense Medical Center’s guidelines for conducting experiments in nonhuman primates.

## Author Contributions

K-HM, W-SH, and C-YC: conceptualization. C-YC: radiosynthesis. Y-YK, C-HC, T-HY, and LF: investigation. SY and K-HM: writing and editing. K-HM, C-YC, and C-JT: funding acquisition and study resources.

## Conflict of Interest

LF was employed by Radiomedix, Inc. The remaining authors declare that the research was conducted in the absence of any commercial or financial relationships that could be construed as a potential conflict of interest.

## Publisher’s Note

All claims expressed in this article are solely those of the authors and do not necessarily represent those of their affiliated organizations, or those of the publisher, the editors and the reviewers. Any product that may be evaluated in this article, or claim that may be made by its manufacturer, is not guaranteed or endorsed by the publisher.

## Supplementary Material

Supplementary Figure 1 Structures of [^123^I]I-ADAM and 4-[^18^F]ADAM.

Supplementary Table 1 Regional specific uptake ratios (SUR) of 4-[^18^F]ADAM in the brain. Data are presented as mean ± SEM; **p* < 0.05, ***p* < 0.01 compared to the control group.

Supplementary Table 2 Recovery of serotonin transport (SERT) availability during the study period. Data are presented as mean ± SEM; **p* < 0.05, ***p* < 0.01, ****p* < 0.005 compared to the control group; #*p* < 0.05 compared to the MDMA group.
