# Supplementary material for: Preliminary Results on the Long-Term Effects of Dextromethorphan on MDMA-Mediated Serotonergic Deficiency and Volumetric Changes in Primates Based on 4-[18F]-ADAM PET/MRI
Source: Front Neurosci. 2022 May 19;16:837194. doi: 10.3389/fnins.2022.837194 (PMC9175743; doi:10.3389/fnins.2022.837194)
Supplement: Supplementary Figure 1 — Structures of [123I]I-ADAM and 4-[18F]ADAM. [file Data_Sheet_1.PDF]

# Suppl.Table 1

## Specific uptake ratio (SUR) of 4-[$^{18}$F]ADAM

| Frontal Cortex | 60M | $p_{vs. Con}$ | 66M | $p_{vs. Con}$ |
|---|---|---|---|---|
| Control | 1.21 ± 0.05 | | 1.12 ± 0.16 | |
| MDMA | 0.97 ± 0.03 | ** | 1.04 ± 0.22 | *NS* |
| DM+MDMA | 1.13 ± 0.01 | *NS* | 1.16 ± 0.12 | *NS* |

| Occipital Cortex | 60M | $p_{vs. Con}$ | 66M | $p_{vs. Con}$ |
|---|---|---|---|---|
| Control | 0.94 ± 0.02 | | 0.92 ± 0.10 | |
| MDMA | 0.82 ± 0.04 | * | 0.82 ± 0.04 | * |
| DM+MDMA | 0.84 ± 0.01 | * | 0.83 ± 0.08 | *NS* |

| Orbitofrontal cortex | 60M | $p_{vs. Con}$ | 66M | $p_{vs. Con}$ |
|---|---|---|---|---|
| Control | 1.21 ± 0.06 | | 1.12 ± 0.16 | |
| MDMA | 1.08 ± 0.05 | *NS* | 1.12 ± 0.21 | *NS* |
| DM+MDMA | 1.13 ± 0.01 | *NS* | 1.19 ± 0.09 | *NS* |

| Cingulate | 60M | $p_{vs. Con}$ | 66M | $p_{vs. Con}$ |
|---|---|---|---|---|
| Control | 1.39 ± 0.05 | | 1.31 ± 0.11 | |
| MDMA | 1.27 ± 0.07 | *NS* | 1.35 ± 0.15 | *NS* |
| DM+MDMA | 1.32 ± 0.01 | *NS* | 1.33 ± 0.10 | *NS* |

| Caudate nucleus | 60M | $p_{vs. Con}$ | 66M | $p_{vs. Con}$ |
|---|---|---|---|---|
| Control | 1.75 ± 0.03 | | 1.65 ± 0.19 | |
| MDMA | 1.50 ± 0.14 | *NS* | 1.65 ± 0.20 | *NS* |
| DM+MDMA | 1.60 ± 0.05 | * | 1.69 ± 0.48 | *NS* |

| Putamen | 60M | $p_{vs. Con}$ | 66M | $p_{vs. Con}$ |
|---|---|---|---|---|
| Control | 1.95 ± 0.04 | | 1.88 ± 0.16 | |
| MDMA | 1.59 ± 0.12 | *NS* | 1.59 ± 0.12 | *NS* |
| DM+MDMA | 1.73 ± 0.06 | * | 1.86 ± 0.33 | *NS* |

| Hippocampus | 60M | $p_{vs. Con}$ | 66M | $p_{vs. Con}$ |
|---|---|---|---|---|
| Control | 1.35 ± 0.03 | | 1.37 ± 0.05 | |
| MDMA | 1.26 ± 0.01 | * | 1.28 ± 0.09 | *NS* |
| DM+MDMA | 1.37 ± 0.04 | # | 1.32 ± 0.04 | *NS* |

| Thalamus | 60M | $p_{vs. Con}$ | 66M | $p_{vs. Con}$ |
|---|---|---|---|---|
| Control | 2.14 ± 0.04 | | 2.09 ± 0.21 | |
| MDMA | 0.19 ± 0.19 | *NS* | 1.87 ± 0.12 | *NS* |
| DM+MDMA | 2.01 ± 0.14 | *NS* | 2.08 ± 0.02 | # |

| Hypothalamus | 60M | $p_{vs. Con}$ | 66M | $p_{vs. Con}$ |
|---|---|---|---|---|
| Control | 1.89 ± 0.11 | | 1.86 ± 0.17 | |
| MDMA | 1.71 ± 0.19 | *NS* | 1.82 ± 0.16 | *NS* |
| DM+MDMA | 1.82 ± 0.04 | *NS* | 1.80 ± 0.03 | *NS* |

| Midbrain | 60M | $p_{vs. Con}$ | 66M | $p_{vs. Con}$ |
|---|---|---|---|---|
| Control | 2.35 ± 0.05 | | 2.27 ± 0.04 | |
| MDMA | 1.71 ± 0.13 | * | 1.82 ± 0.13 | * |
| DM+MDMA | 2.24 ± 0.18 | *NS* | 2.20 ± 0.09 | *NS* |

| Amygdala | 60M | $p_{vs. Con}$ | 66M | $p_{vs. Con}$ |
|---|---|---|---|---|
| Control | 1.69 ± 0.04 | | 1.70 ± 0.03 | |
| MDMA | 1.37 ± 0.13 | * | 1.58 ± 0.09 | * |
| DM+MDMA | 1.67 ± 0.08 | *NS* | 1.76 ± 0.04 | # |

# Suppl.Table 2

## Recovery rate (%) of SERT availability

| Frontal Cortex | 60M | $p_{vs. Con}$ | $p_{vs. MDMA}$ | 66M | $p_{vs. Con}$ | $p_{vs. MDMA}$ |
|---|---|---|---|---|---|---|
| Control | 100.00% | | | 100.00% | | |
| MDMA | 86.65% ± 2.94% | *** | | 78.89% ± 7.74% | * | |
| DM+MDMA | 93.64% ± 0.88% | * | NS | 88.01% ± 4.54% | * | NS |

| Occipital Cortex | 60M | $p_{vs. Con}$ | $p_{vs. MDMA}$ | 66M | $p_{vs. Con}$ | $p_{vs. MDMA}$ |
|---|---|---|---|---|---|---|
| Control | 100.00% | | | 100.00% | | |
| MDMA | 83.44% ± 0.45% | *** | | 90.56% ± 7.13% | NS | |
| DM+MDMA | 93.64% ± 10.00% | *** | NS | 92.66% ± 3.84% | * | NS |

| Orbitofrontal cortex | 60M | $p_{vs. Con}$ | $p_{vs. MDMA}$ | 66M | $p_{vs. Con}$ | $p_{vs. MDMA}$ |
|---|---|---|---|---|---|---|
| Control | 100.00% | | | 100.00% | | |
| MDMA | 80.39% ± 2.94% | *** | | 90.11% ± 3.90% | * | |
| DM+MDMA | 91.38% ± 1.99% | ** | NS | 94.46% ± 9.87% | NS | NS |

| Cingulate | 60M | $p_{vs. Con}$ | $p_{vs. MDMA}$ | 66M | $p_{vs. Con}$ | $p_{vs. MDMA}$ |
|---|---|---|---|---|---|---|
| Control | 100.00% | | | 100.00% | | |
| MDMA | 86.33% ± 4.96% | * | | 92.11% ± 4.72% | * | |
| DM+MDMA | 94.81% ± 3.86% | NS | NS | 97.55% ± 8.35% | NS | NS |

| Caudate_nucleus | 60M | $p_{vs. Con}$ | $p_{vs. MDMA}$ | 66M | $p_{vs. Con}$ | $p_{vs. MDMA}$ |
|---|---|---|---|---|---|---|
| Control | 100.00% | | | 100.00% | | |
| MDMA | 84.72% ± 6.63% | * | | 86.86% ± 5.06% | * | |
| DM+MDMA | 91.48% ± 4.49% | * | NS | 88.93% ± 3.29% | ** | NS |

| Putamen | 60M | $p_{vs. Con}$ | $p_{vs. MDMA}$ | 66M | $p_{vs. Con}$ | $p_{vs. MDMA}$ |
|---|---|---|---|---|---|---|
| Control | 100.00% | | | 100.00% | | |
| MDMA | 82.78% ± 5.58% | * | | 83.43% ± 3.29% | ** | |
| DM+MDMA | 89.40% ± 4.82% | * | NS | 98.11% ± 9.18% | NS | NS |

| Hippocampus | 60M | $p_{vs. Con}$ | $p_{vs. MDMA}$ | 66M | $p_{vs. Con}$ | $p_{vs. MDMA}$ |
|---|---|---|---|---|---|---|
| Control | 100.00% | | | 100.00% | | |
| MDMA | 93.47% ± 2.09% | * | | 93.45% ± 3.19% | * | |
| DM+MDMA | 98.41% ± 0.51% | * | NS | 94.19% ± 0.22% | * | NS |

| Thalamus | 60M | $p_{vs. Con}$ | $p_{vs. MDMA}$ | 66M | $p_{vs. Con}$ | $p_{vs. MDMA}$ |
|---|---|---|---|---|---|---|
| Control | 100.00% | | | 100.00% | | |
| MDMA | 83.21% ± 7.18% | * | | 78.83% ± 2.40% | *** | |
| DM+MDMA | 89.55% ± 6.08% | NS | NS | 99.31% ± 8.87% | NS | # |

| Hypothalamus | 60M | $p_{vs. Con}$ | $p_{vs. MDMA}$ | 66M | $p_{vs. Con}$ | $p_{vs. MDMA}$ |
|---|---|---|---|---|---|---|
| Control | 100.00% | | | 100.00% | | |
| MDMA | 93.47% ± 2.09% | * | | 88.06% ± 3.75% | * | |
| DM+MDMA | 89.14% ± 4.41% | * | NS | 97.22% ± 8.05% | NS | NS |

| Midbrain | 60M | $p_{vs. Con}$ | $p_{vs. MDMA}$ | 66M | $p_{vs. Con}$ | $p_{vs. MDMA}$ |
|---|---|---|---|---|---|---|
| Control | 100.00% | | | 100.00% | | |
| MDMA | 82.34% ± 6.06% | * | | 91.68% ± 2.43% | * | |
| DM+MDMA | 94.06% ± 5.03% | NS | NS | 96.69% ± 1.04% | * | NS |

| Amygdala | 60M | $p_{vs. Con}$ | $p_{vs. MDMA}$ | 66M | $p_{vs. Con}$ | $p_{vs. MDMA}$ |
|---|---|---|---|---|---|---|
| Control | 100.00% | | | 100.00% | | |
| MDMA | 81.13% ± 2.39% | *** | | 92.91% ± 2.43% | * | |
| DM+MDMA | 91.81% ± 4.47% | * | NS | 101.77% ± 0.34% | ** | # |

**Suppl. Fig.1**

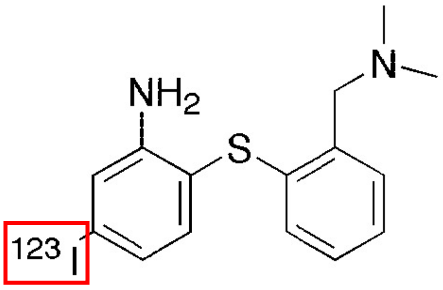


$[^{123}I]I$-ADAM

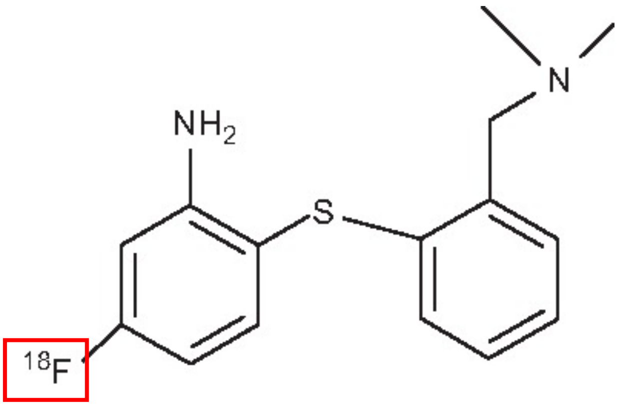


4-$[^{18}F]$ADAM
